# Perspectives on Adipose Tissue, Chagas Disease and Implications for the Metabolic Syndrome

**DOI:** 10.1155/2009/824324

**Published:** 2009-07-26

**Authors:** Fnu Nagajyothi, Mahalia S. Desruisseaux, Linda A. Jelicks, Fabiana S. Machado, Streamson Chua, Philipp E. Scherer, Herbert B. Tanowitz

**Affiliations:** ^1^Department of Pathology, Albert Einstein College of Medicine, 1300 Morris Park Avenue, Bronx, NY 10461, USA; ^2^Department of Medicine, Albert Einstein College of Medicine, 1300 Morris Park Avenue, Bronx, NY 10461, USA; ^3^Department of Physiology & Biophysics, Albert Einstein College of Medicine, 1300 Morris Park Avenue, Bronx, NY 10461, USA; ^4^Department of Biochemistry and Immunology, Institute of Biological Sciences, Federal University of Minas Gerais, Pampula, Belo Horizonte 3127-901, Brazil; ^5^Touchstone Diabetes Center, Departments of Internal Medicine and Cell Biology, University of Texas Southwestern, Dallas, TX 75390, USA

## Abstract

The contribution of adipose tissue an autocrine and endocrine organ in the pathogenesis of infectious disease and metabolic syndrome is gaining attention. Adipose tissue and adipocytes are one of the major targets of *T. cruzi* infection. Parasites are detected 300 days postinfection in adipose tissue. Infection of adipose tissue and cultured adipocytes triggered local expression of inflammatory mediators resulting in the upregulation of cytokine and chemokine levels. Adipose tissue obtained from infected mice display an increased infiltration of inflammatory cells. Adiponectin, an adipocyte specific protein, which exerts antiinflammatory effects, is reduced during the acute phase of infection. The antiinflammatory regulator peroxisome proliferator activated receptor-*γ* (PPAR-*γ*) is downregulated in infected cultured adipocytes and adipose tissue. *T. cruzi* infection is associated with an upregulation of signaling pathways such as MAPKs, Notch and cyclin D, and reduced caveolin-1 expression. Adiponectin null mice have a cardiomyopathy and thus we speculate that the *T. cruzi*-induced reduction in adiponectin contributes to the *T. cruzi*-induced cardiomyopathy. While *T. cruzi* infection causes hypoglycemia which correlates with mortality, hyperglycemia is associated with increased parasitemia and mortality. The *T. cruzi*-induced increase in macrophages in adipose tissue taken together with the reduction in adiponectin and the associated cardiomyopathy is reminiscent of the metabolic syndrome.

## 1. Introduction

Diseases caused by nematodes and protozoa have been reported to be associated with nutritional deficiencies, wasting, and diabetes. An association between human *Trypanosoma cruzi* infection (Chagas disease) and obesity and diabetes has been suspected. 

Chagas disease is an important cause of morbidity and mortality in Latin America where 10% to 30% of infected individuals eventually succumb to the chronic manifestations such as cardiomyopathy and/or mega syndromes. This infection is also an opportunistic infection in those individuals who are immunosuppressed including those with HIV/AIDS. Although the pathogenesis of Chagas disease has been investigated by many laboratories the role of the adipocyte and of adipose tissue has been ignored.

## 2. The Adipocyte and Adipose Tissue: General Considerations

The contribution of the adipocyte or fat cell to the pathogenesis of diabetes, obesity and the metabolic syndrome is well recognized [1–5]. Adipose tissue is not a mere storage compartment for triglycerides. Adipocytes or fat cells are active endocrine cells that play an important role in energy homeostasis and the immune system [6]. Adipocytes influence systemic lipid homeostasis through the production and release of adipocyte-specific and adipocyte-enriched hormonal factors, inflammatory mediators such as cytokines, chemokines, and extracellular matrix components also known as adipokines. The strong proinflammatory potential of adipose tissue suggests an important role in the systemic innate immune response. 

Although the most prominent cell type in adipose tissue is the adipocyte, there are other cell types such as fibroblasts, endothelial cells, smooth muscle cells and inflammatory cells. Different adipose tissue depots display distinct gene expression patterns and vary widely in size and proximity to neighboring organs. Although differences exist between the different fat pads, the depots share similarity with respect to their ability to store lipids and secrete adipose tissue-derived hormones. Adipose tissue stores lipid in the form of triglycerides and also stores mostly nonesterified cholesterol on the surface of the lipid droplets that represent specialized organelles inside the adipocyte. 

The potential endocrine function of adipose tissue was first appreciated with the report that the serine protease adipsin was secreted by the cultured 3T3-L1 adipocytes [7]. Subsequently, several additional adipokines have been discovered [8, 9]. These adipokines contribute to the regulation of energy homeostasis through effects on both central and peripheral tissues. Several of these adipokines also contribute to nonmetabolic processes in the body emphasizing the fact that adipokines participate in the coordination of multiple physiological functions in a variety of tissues. The most adipocyte-specific adipokine is adiponectin. Other adipokines can also be synthesized by tissues other than adipose tissue and/or by cells other than adipocytes. 

Systemic energy homeostasis is maintained by competing effects of a number of different hormonal factors, some of which originate in adipose tissue. These adipocyte-derived factors (adipokines), influence processes such as food intake, energy expenditure and insulin sensitivity in a variety of tissues. Two adipokines, resistin and adiponectin have opposite effects on whole-body glucose homeostasis [1, 10]. Pharmacological doses of recombinant resistin hyper-activate gluconeogenesis through decreased hepatic insulin sensitivity. 

Adiponectin, a hormone exclusively produced by the adipocytes, is a 30-kDa molecule with three defined domains. Both intracellular and extracellular adiponectin exists in three different higher order complexes: high molecular weight form (HMW; 12 to 36 mer), and low molecular weight form (hexamer and trimeric). The different complexes exert distinct functions, and the ratio of HMW to the other forms serves as an independent predicting factor for metabolic disorders. The total level and HMW ratio are decreased in obese patients and obese mouse models. This suggests that adiponectin, especially the HMW form, may be involved in obesity-related disorders. It has been demonstrated that adiponectin increases insulin sensitivity by inhibiting hepatic glucose output. Lower levels of circulating adiponectin are associated with increased susceptibility to a variety of diseases of metabolic dysfunction including diabetes, hypertension and obesity. 

There is an association between circulating adiponectin levels and various metabolic parameters regulating insulin sensitivity in many different patient populations. For example, there is a decrease in plasma adiponectin concentration in obese humans [11]. Other studies showed that this finding could be extended to obese rodents and other animal models. The pattern of decreased adiponectin secretion with increasing adiposity has been well recognized. There is a reduction in the levels of adiponectin in diabetics with coronary artery disease compared with diabetics without coronary artery disease and the adiponectin levels in serum are negatively correlated with basal metabolic rate, plasma glucose and insulin and serum triglycerides [12]. Interestingly, even a relatively moderate weight loss led to a significant increase in circulating adiponectin levels in both diabetics and nondiabetics. In morbidly obese individuals [13] undergoing gastric partition surgery, a decrease in basic metabolic rate was noted and fasting glucose and insulin levels were associated with a similar increase in circulating levels of adiponectin together with an increase in insulin sensitivity. 

Adiponectin expression and/or secretion may be directly or indirectly regulated by plasma insulin levels. Previous studies have demonstrated that insulin treatment of 3T3-L1 adipocytes results in significantly decreased adiponectin expression [14], and serum adiponectin levels are inversely proportional to fasting insulin levels. A corollary is that a feedback inhibitory pathway must exist that downregulates expression and secretion of adiponectin in obesity. Intra-abdominal and mesenteric fat pads (central fat pads) are predominant sources of systemic adiponectin in the lean state and the production of adiponectin in the obese state is reduced.

## 3. Adiponectin, Inflammation, and Heart Disease

 Individuals with the highest levels of adiponectin had a reduced risk of myocardial infarction compared with those with the lowest adiponectin levels. Animal models have corroborated these observations, demonstrating the importance of adiponectin for preventing diet-induced progression of atherosclerosis. It should be noted, however, that the mechanism of the antiatherosclerotic activity of adiponectin has not been entirely elucidated. It has been hypothesized that adiponectin has inflammatory-modulating activities, and clinical studies have demonstrated an inverse relationship between adiponectin levels and serum markers of inflammation [15, 16]. It is reported that antiinflammatory effects on both endothelium and macrophages with the physiologically relevant full-length form exist [4, 9]. It is unclear whether adiponectin itself has antiinflammatory properties. However, adiponectin production by adipose tissue can be inhibited by systemic inflammation, at least under some circumstances. 

Adiponectin production by adipocytes in culture is inhibited by inflammatory cytokines such as TNF-*α* [9, 17] and this inhibition may be mediated in part by NF*κ*B signaling. I*κ*B Kinase inhibition leads to increased plasma adiponectin levels and an improvement in systemic insulin sensitivity [18]. The antiinflammatory activity of adiponectin may be mediated by its principal signaling target, the AMP-activated protein kinase (AMPK). 

Chemokines positively control the secretion of leptin suggesting a role for chemokines in the regulation of adipose tissue and suggest a novel therapeutic basis for the treatment of obesity, diabetes and cachexia [19]. A high-fat diet increases the expression of inflammatory genes including early induction of MCP-1 and MCP-3 [20]. The antiatheromatous effects of adiponectin may be mediated by antiinflammatory activities acting directly on the vasculature. For example, Okamoto et al. [21] recently reported that adiponectin inhibits the production of CXCR-3 chemokine ligands in macrophages and causes a reduction in T-lymphocyte recruitment. 

Adiponectin contributes toward protection against cardiac hypertrophy in cardiac overload states including hypertension, hypertrophic cardiomyopathy, and ischemic heart disease. In mice, adiponectin protects against myocardial ischemia-reperfusion injury and overload as well as adrenergically-induced cardiac myocyte hypertrophy, by inhibiting hypertrophic signals via AMPK [22–24]. Notably, adiponectin null mice have a cardiomyopathic phenotype [22, 25]. Thus, the current information is consistent with the idea that adiponectin is antiinflammatory and reduced levels of adiponectin are proinflammatory.

## 4. Adipose Tissue Adipocytes and Infection

The relationship between adipocytes and infectious agents has only recently received attention. For example, there have been investigations into the infectious etiologies of obesity [26]. A role for adipose tissue in infection was also underscored by the work of the Scherer laboratory who demonstrated that injection of LPS into mice that were rendered fatless by manipulation of the apoptosis pathway did not cause immediate death of mice as they did in control mice with a normal component of adipose tissue [27]. These observations suggested that the inflammatory mediators resulting from adipose tissue play an important role in the inflammatory response to infection. One of the most intensively investigated areas in the interface between infection and adipose tissue has been in HIV/AIDS [28–31].

## 5. Trypanosoma cruzi Infection

The relationship between blood sugar and *T. cruzi* infection has undergone limited investigation. Studies from our laboratory demonstrated that when mice with chemical-induced diabetes were infected with *T. cruzi*, they had a higher parasitemia and mortality and this was reversed by treatment with insulin [32]. *T. cruzi*-infected obese diabetic *db*/*db* mice also displayed a high parasitemia and increased mortality [32]. The underlying pathophysiological mechanisms of these observations remain unknown. Recently**, **we infected mice and observed hypoglycemia which generally correlated with mortality [33]. Interestingly, the metabolic response to bacterial sepsis is hyperglycemia, insulin resistance, profound negative nitrogen balance, and diversion of protein from skeletal muscle to splanchnic tissues. Thus, the response to *T. cruzi* infection differs from that generally observed in bacterial sepsis. It is possible that there is an effect on glucose metabolism due to invasion of the liver by the parasite. During acute infection, glucose levels in all the *T. cruzi*-infected mice were below the levels measured for the control mice. Even though the baseline glucose levels in the infected animals were lower, the oral glucose tolerance test indicated relatively normal ability to clear the ingested glucose despite the high degree of inflammation associated with this infection. 

Infection of mice with *T. cruzi* results in hypoglycemia. This may be a result of increased uptake of glucose by the parasite [34]. Another explanation is an effect on glucose metabolism due to invasion of the liver by the parasite. The precise mechanism(s) for hypoglycemia during the acute *infection* is not known. 

Adiponectin levels were significantly reduced during *T. cruzi* infection of several different strains of mice (see [10] and unpublished observations). Reduced levels of adiponectin are usually associated with insulin resistance, hyperglycemia, and obesity, that is, the metabolic syndrome. Decreased levels of adiponectin are observed in the setting of some types of inflammation and cardiovascular disease. Acute inflammation induced by endotoxemia does not affect adiponectin levels [18]. The infection-induced hypoglycemia cannot be readily explained by changes in adiponectin. Thus, this is an interesting example of a physiologically relevant condition that combines hypoglycemia and normal glucose tolerance with significantly reduced adiponectin levels. The decreased insulin levels observed during infection in the mouse model of *T. cruzi* infection are consistent with a physiological response to the very low glucose levels. Leptin levels were also significantly reduced in infected mice compared to controls [33]. Resistin levels, another fat cell-specific secretory factor with insulin-desensitizing properties, were not affected by infection [33]. Levels of plasminogen activator inhibitor-1, which is also prominently expressed in adipocytes, were completely unaffected by infection. Proinflammatory markers such as cytokines and chemokines were markedly elevated in the adipose tissue of acutely infected mice and persisted into the chronic phase. 

Initially, the significant decrease in leptin levels was surprising since the infected mice gained more weight than the control mice. Magnetic resonance imaging studies as well as the body composition studies using an ECHO magnetic resonance spectrometry (MRS) body composition instrument revealed a decrease in abdominal adipose tissue. Mice that had marked right ventricular dilation had a greater loss of fat depots. The weight gain in infected mice appeared to be related to edema, which may have been the consequence of right-sided heart failure [33]. 

CD-1 and FVB mice infected with the Brazil strain of *T. cruzi* displayed a reduction in plasma levels of adiponectin suggesting that infection of adipocytes may also have consequences on other proteins synthesized in adipose tissue. The level of adiponectin in adipose tissue was also reduced during acute infection in a number of fat pads known to be important sources of adiponectin. During acute infection, the acute-phase reactants *α*-1 acid glycoprotein and SAA3, which are expressed in adipocytes, were upregulated. Consistent with the infection-induced increase in inflammatory mediators (cytokines and chemokines) there was a concomitant reduction in adiponectin and peroxisome proliferator-activated receptor-*γ* (PPAR-*γ*). Both of these proteins are negative regulators of the inflammatory pathway. 

We also demonstrated by qPCR that *T. cruzi * DNA in adipose tissue 300 days post infection was at the same levels as in the heart [33]. This observation suggests that the adipocyte may serve as an important target for *T. cruzi* and in chronic Chagas disease adipocytes may represent an important long-term reservoir for parasites from which relapse of infection can occur. 

**Figure 1:**
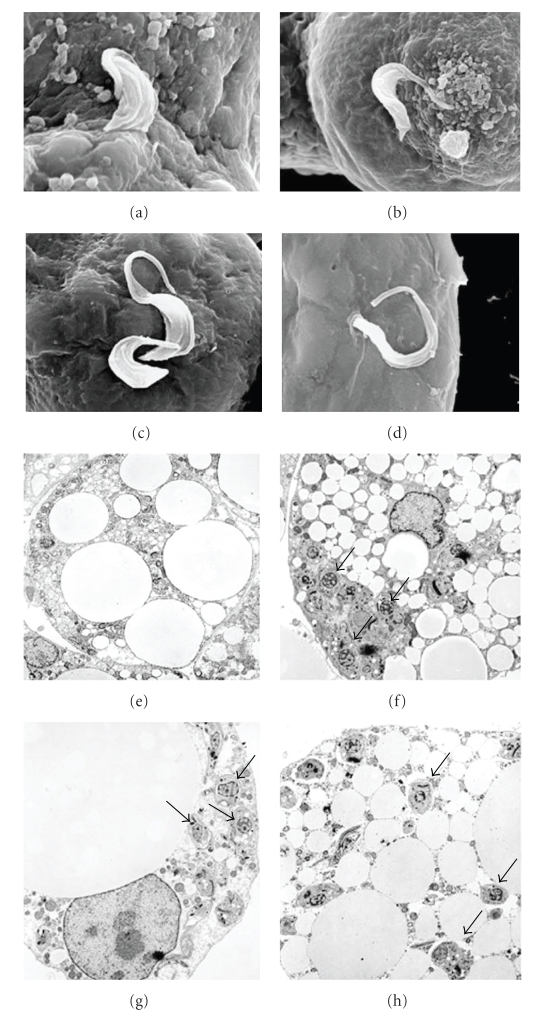
(a)–(d) Scanning electron micrograph of trypomastigotes of *T. cruzi * invading adipocyte. (e) A transmission electron micrograph of an uninfected adipocyte. (f)–(h) Electron micrographs of infected adipocytes. The parasites (arrows) are in close proximity to the lipid droplets (with permission of the Journal of Biological Chemistry).

Next our laboratory performed in vitro studies to evaluate the role of the adipocyte in *T. cruzi* infection in a model system devoid of many other cell types ordinarily found in adipose tissue. We were not the first group to observe *T. cruzi* in adipocytes [35] but we were the first group to investigate this relationship in detail [33, 36]. Adipocytes infected for 96 hours maintain their integrity and intracellular amastigotes are observed as monitored by electron microscopy (Figure 1). *T. cruzi* infection of cultured adipocytes displays an inflammatory phenotype. For example, there was increased expression of chemokines such as CCL2, CCL3, CCL5, and CXCL10 and the cytokines TNF-*α*, IL-10, and interferon-*γ*. The expression of SAT3, an important downstream mediator of cytokine signaling, was increased as well. Toll-like receptors (TLRs) TLR-2 and -9 reported to be activated during *T. cruzi* infection of other cell types were also upregulated in adipocytes *T. cruzi* infection also activated ERK and p38 MAPK. 

*T. cruzi* infection of cultured adipocytes resulted in increased expression of cyclin D1. Cyclin D1 is generally associated with cell proliferation but cultured adipocytes are usually terminally differentiated. The increased expression of cyclin D1 is important because it is upregulated by ERK and inversely regulated by caveolin-1 [37]. In infected cultured adipocytes, we demonstrated that infection resulted in a reduction in the expression of caveolin-1 and activation of ERK; both of these events increase the expression of cyclin D1. The reduction in caveolin-1 expression has also been demonstrated to be associated with an increased proinflammatory cytokine response [38, 39]. Interestingly, infection activates the Notch pathway which regulates the expression of cyclin D1 [40]. 

PPAR-*γ* is expressed in adipose tissue and similar to adiponectin is antiinflammatory [41, 42]. As noted, the reduction in the level of adiponectin is associated with an increase in inflammation [9]. In addition, there is an inverse relationship between PPAR-*γ* and inflammation as well as between PPAR-*γ* and cyclin D1 [43]. It has been demonstrated that increased expression of cyclin D1 is associated with a reduction in PPAR-*γ*. Recent evidence suggests a similar relationship between adiponectin and PPAR-*γ* [41, 42]. Our observations clearly demonstrated that *T. cruzi* infection resulted in a reduction in the expression of adiponectin and PPAR-*γ* and an increase in the expression of cyclin D1 and inflammatory mediators.

Infection also results in increased expression of PI-3 kinase and activation of AKT, suggesting that this infection may induce components of the insulin/IGF-1 receptor cascade. The upregulation of proinflammatory pathways is generally associated with a downregulation of the insulin signal transduction pathway [44, 45]. It is not clear what is responsible for this phenomenon, but it can be observed with a high degree of reproducibility in these cells. *T. cruzi* invasion is facilitated through the activation of host cell PI-3 kinases in mammalian cells [46]. Human Schwann cells infected with *T. cruzi* suppressed host-cell apoptosis through trans sialidase activity via the PI3k/AKT pathway, suggesting a role for PI3K/AKT in the pathogenesis of Chagas disease [47]. Despite the upregulation of some of the components of the pathway, there were no differences with respect to a dose response to insulin in infected cells (unpublished observations). It remains to be determined whether other pathways influenced by insulin may be affected, such as events leading to differences in the rate of lipid accumulation or lipolysis. *T. cruzi* is likely to have an impact on lipid pathways in vivo, yet these issues have not been examined to date.

Our findings are significant since there is a positive correlation between inflammation and insulin resistance. However, the infection of adipocytes with a parasite that resides intracellularly is different from exposing adipocytes to conventional proinflammatory stimuli such as endotoxin. The continued intracellular presence of the parasites clearly has a differential effect on insulin sensitivity, perhaps by lowering the levels of one of the critical lipid mediators of insulin resistance. 

Fat and glucose metabolism are interrelated and dysregulated in *T. cruzi* infection. Adipocytes and adipose tissue represent an important target of and reservoir for infection. This is a reservoir from which parasites can be reactivated during periods of immunosuppression. In addition, infection of the adipocyte and adipose tissue creates an inflammatory phenotype that affects a variety of metabolic processes. Furthermore, the reduction in the expression of adiponectin and persistent inflammation and PPAR-*γ* perpetuate the inflammatory phenotype. Since adiponectin null mice have a cardiomyopathic phenotype it is tempting to suggest that the reduction in adiponectin and PPAR-*γ* contributes to the cardiomyopathy of Chagas disease.
